# Adherence to Public Health England (PHE) guidance for the use of personal protective equipment (PPE) in north Wales mental health unit- a regional audit

**DOI:** 10.1192/bjo.2021.838

**Published:** 2021-06-18

**Authors:** Asha Dhandapani, Sathyan Soundararajan, Alberto Salmoiraghi, Shona Ginty, Tajnin Mitu, Justina Akinlua, Catrin Thomas, Rahul Malhotra, Zeenish Azhar, Haseeb Bhutta, Hanani Taib, Nikhil Gauri Shankar, Vikram Bhangu, Gathoni Kamau, Elizabeth Chamberlain, Anna Mackenzie, Henrik PAHLEN, Hannah Lock, Aniis Rymansaib, Pauline Mclean, Rodrigo Trujillo, Manjula Simiyon, Adam Chappell, Agnieszka Gross, Gaynor Gaskell

**Affiliations:** BCUHB

## Abstract

**Aims:**

To ensure that the PPE guidance is strictly adhered to.

To ensure that patient care is not compromised.

To help us in areas of need in order to educate the staff regarding the techniques of PPE and thus ensure patient and staff safety and care during the pandemic.

**Method:**

Novel coronavirus 2019 was first described in December 2019 in Wuhan in China. Since those initial few cases, it has rapidly proliferated to a global pandemic, putting an inordinate amount of strain on healthcare systems around the world. We believe that the technique of donning and doffing if followed as per PHE guidelines would be of help in both preventing the infection and improve the care and safety of both patients and staff.

This Audit includes both In-patient and Out-patient units in Psychiatric services across North Wales. Data were collected from 19 units out of 39. We observed covertly 325 staff members belonging to various cadres. Apart from the Donning and Doffing techniques, we also observed the availability of designated areas for this purpose and the availability of PPE as well.

Data collection was by junior and senior doctors from various sites of the mental health unit in North Wales. A proforma was provided, the standards were based on PHE guidelines.

**Result:**

It was noted that just about 50% of the staff followed donning as per guidance. Amongst all three sites, the Central team showed a better adherence with 85% of them donning PPE correctly. whereas only 22% adhered to donning in the West team.

Only 21% of them managed to doff PPE as per guidance amongst all 3 centres in North Wales.

It was also noted that there are no designated areas to Don and Doff in outpatient units. Staff, in general, seem to not adhere to the guidance of utilising a mask, especially when within 2 meters distance of other staff.

**Conclusion:**

We will be presenting the Audit at the regional meeting. After discussion with the infection prevention control team and Health and safety lead, we intend to improvise the wards with designated areas for donning and doffing. Teaching sessions for the staff in all three sites, reminders in various areas of the community mental health units and inpatient units.

We are hoping that these recommendations will help us in achieving our aim of health and safety during this pandemic.

